# Supplementary material for: Network-specific metabolic cost of functional connectivity in the human brain
Source: bioRxiv. 2026 Sep 10:2026.09.09.750304. Preprint. [Version 1] doi: 10.64898/2026.09.09.750304 (PMC13622659; doi:10.64898/2026.09.09.750304)
Supplement: Supplementary file 1 [file media-1.pdf]

SUPPLEMENTARY MATERIALS

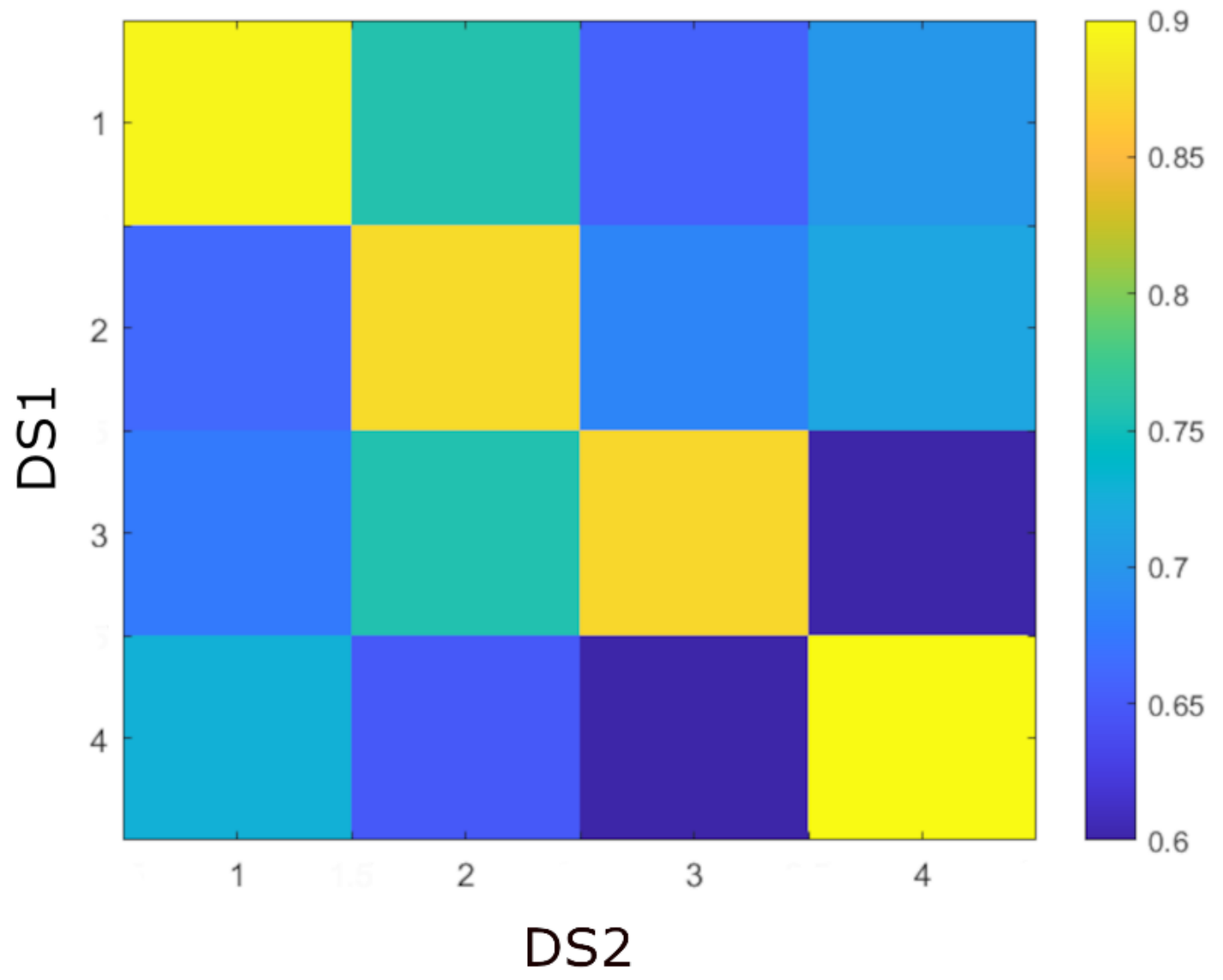

Supplementary Figure 1: Correlation matrix of states' centroids between datasets

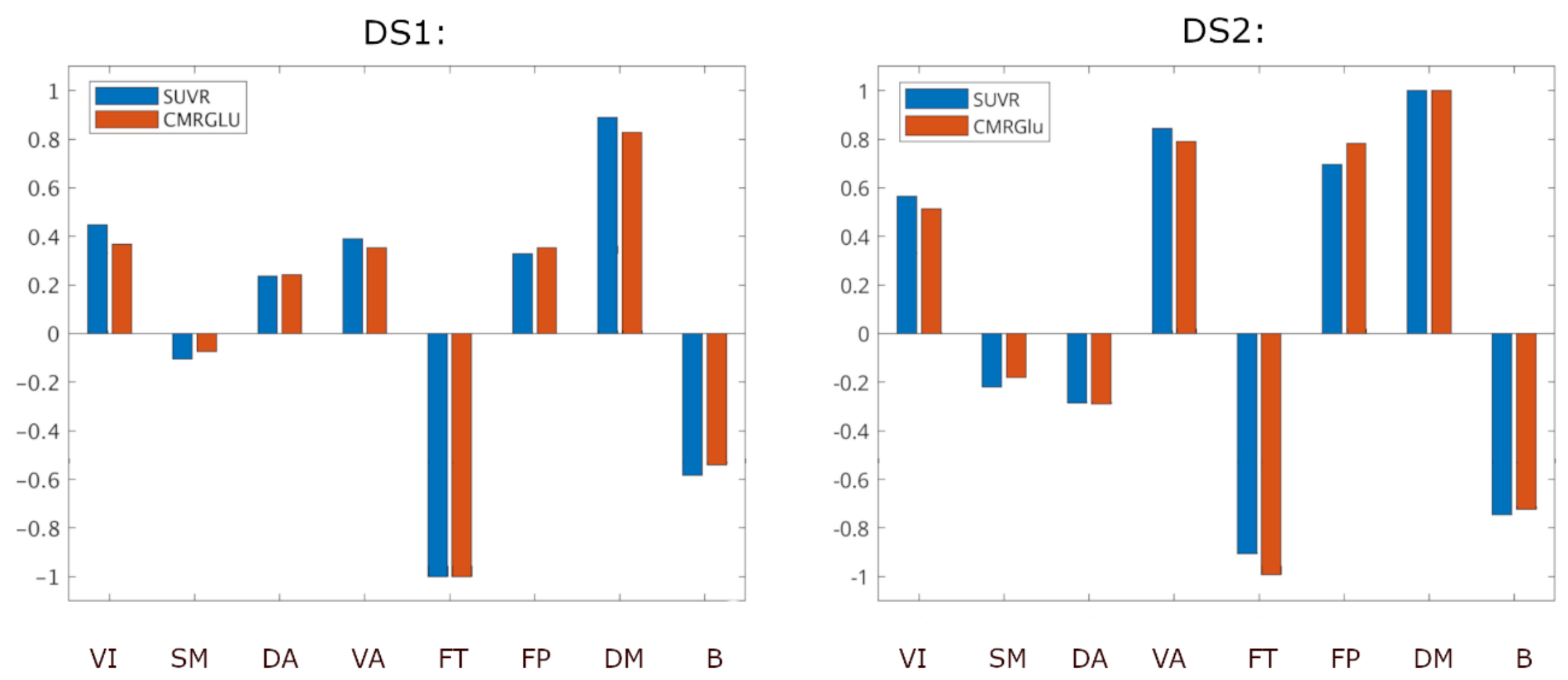

Supplementary Figure 2: Normalized resting-state metabolic costs of FC calculated in terms of CMRGLU and SUVR to enable comparison
